# Strengthening the “P” in Maternal and Perinatal Death Surveillance and Response in Bungoma county, Kenya: implications for scale-up

**DOI:** 10.1186/s12913-019-4431-4

**Published:** 2019-08-30

**Authors:** Sarah Bandali, Camille Thomas, Phidelis Wamalwa, Shanti Mahendra, Peter Kaimenyi, Osman Warfa, Nicole Fulton

**Affiliations:** 1 Options Consultancy Services, 2nd Floor, St Magnus House, 3 Lower Thames Street, London, EC3R 6HD UK; 2 Kenya Ministry of Health, Afya House, Cathedral Road, P.O. Box:30016–00100, Nairobi, Kenya

**Keywords:** Perinatal death reviews, Perinatal mortality, Maternal and perinatal death review and response systems, Kenya

## Abstract

**Background:**

This paper examines perinatal death reporting and reviews in Bungoma county, Kenya, where substantial progress has been made, providing important insights for wider scale up to other contexts.

**Methods:**

Quantitative methods were used to analyse trends in perinatal death reporting and reviews between 2014 and 2017 throughout Kenya based on data from the District Health Information System. Qualitative methods helped further understand the success of perinatal death reporting and review in Bungoma county through focus group discussions and individual interviews at 5 hospitals and 1 health centre. Thematic analysis was used to draw out codes for the analysis.

**Results:**

Only 13 of the 47 counties in Kenya conduct perinatal death reviews. In 2017, the year after the perinatal death review system was introduced, only 3.6% of perinatal deaths were reviewed in Kenya. Bungoma county has made the greatest strides in Kenya, reviewing 59% of the perinatal deaths that occurred within the county in 2017. Bungoma accounted for 51% of all the perinatal deaths reviewed in Kenya. Factors contributing to the success in Bungoma include harmonisation of facility based perinatal reporting tools with the national level; prioritising the need to document and report mortalities; tailoring continual medical education and supportive supervision visits to needs identified from the review; and better documentation and referral processes. Supportive management and administrative staff have also helped drive forward implementation of actions and increased health staff motivation to reduce perinatal deaths and improve quality of care.

**Conclusions:**

Successful implementation of perinatal death reviews requires clear delineation of roles and responsibilities for action, which are routinely monitored to track implementation progress. As in other low-income settings, Bungoma county has demonstrated that in Kenya, perinatal death reviews can be effectively implemented and sustained, through a focus on learning, solution-oriented responses, influencing those in a power to act, accountability for results, and observable quality of care improvements.

## Background

Globally, there were 2.6 million newborn deaths in 2016 with 7000 newborns dying each day from pregnancy related complications [1, 2]. The majority of these deaths are preventable through the provision of quality of care during pregnancy and childbirth [2]. In Kenya, the neonatal mortality rate is 22 deaths per 1000 live births (2010–2014), with over 55% of these deaths in the first month of life [3]. Although neonatal mortality has decreased over time to 33 deaths per 1000 live births from 1999 to 2003, significant acceleration is needed for the country to meet its Sustainable Development Goal (SDG) of reducing neonatal mortality to below 12 deaths per 1000 live births by 2030 [4].

Estimates from the Kenya District Health Information System (DHIS2) in 2017 show that show that approximately 1,700,000 women get pregnant in Kenya each year, and about 4% of these women are from Bungoma county. 74% of these women in Kenya register for antenatal care, and approximately 53% of estimated pregnant women (or 73% of those who register for antenatal) gave birth at a facility in 2017. While facility deliveries are increasing, some deliveries continue to happen at home, where reporting has not been streamlined into the main data system. According to most recent Kenya Demographic Health Survey (DHS) 2014, 37.4% of the total deliveries nationally were home deliveries. This information was however not available at county level due to sample size.

The Kenya DHS 2014 estimated a national maternal mortality ratio (MMR) of 362 per 100,000 livebirths (no county specific analysis available). Estimates from DHIS2 show that facility based MMR in Kenya increased from 109 per 100,000 livebirths in 2014, to 124 per 100,000 livebirths in 2017; and from 100 to 129 per 100,000 live births in Bungoma County during the same time period. This could be related to an increase of deliveries taking place at facilities and/or the prolonged health care worker industrial action strikes in 2017. In Bungoma county, the stillbirth rate is declining, from 24 to 17 deaths per 1000 total births in 2014 and 2017 respectively (DHIS2). Perinatal mortality rates have also been steadily reducing from 32 to 24 deaths per 1000 total births in 2014 and 2017 respecitvely in Bungoma (DHIS2).

Maternal and Perinatal Death Surveillance and Response (MPDSR) systems provide information on the magnitude of and factors leading to these deaths, with the aim of identifying preventable actions to avoid further mortalities [5, 6]. MPDSR takes a systems wide approach to understanding and responding to deaths with observed effects on policy and quality of care improvements when successfully implemented, even in low resource settings [7, 8]. It is vital that information arising from death reviews are acted upon if mortality outcomes are to improve.

Perinatal death reviews have been used in a number of settings to measure, identify and take action to reduce perinatal mortality [9–11]. Of the 51 countries that completed the Every Newborn Action Plan Tracking Tool in 2016, only 23 countries began implementation of perinatal death reviews [12].

One meta-analysis of before and after effects found a 30% decline in perinatal mortality in low and middle-income countries after perinatal death reviews were implemented [11], enforcing its value in understanding causes of death, improving quality of care and preventing avoidable deaths [8].

In 2016, perinatal death was added as a component to the national MDSR guidelines in Kenya [13]. However, the implementation of perinatal death reviews has posed a major challenge to implementation. The high volume of perinatal deaths makes reviewing each death overwhelming to overworked health care providers. Normative acceptance and lack of recognition of early infant deaths minimise the perceived value of reporting a death, particularly if it occurred in the community. Finally, there are gaps in documentation forms and tools for perinatal reporting that make it difficult to capture and collate vital information.

In Kenya, the national government is responsible for policy formulation, health legislation and regulation, while health service planning and delivery is a mandate of the county government. Bungoma is one of Kenya’s 47 counties, and is situated in the Western region, which experiences one of the lowest proportions of women delivering with a skilled birth attendant (48%) and a high perinatal mortality rate (29%) [14]. In Bungoma county, substantial progress has been made in strengthening perinatal death reviews and taking action to prevent further perinatal deaths. The UKAID supported Maternal and Newborn Health Improvement (MANI) project contributed to this progress through training sub-county health management teams (CHMTs) and facility staff in six out of the county’s ten sub-counties in Bungoma on the national MPDSR guidelines. Similar training was provided by another development partner in the other four sub-counties of Bungoma. In all sub-counties, MPDSR review committees met regularly to review deaths. Facilities that had high volumes of clients usually recorded higher numbers of mortalities. In these cases, the MANI project provided technical support to the MPDSR committees to conduct in-depth reviews of a sample of perinatal deaths. Smaller facilities with fewer mortalities received technical support to routinely review all perinatal deaths.

This paper examines perinatal death reporting and reviews nationally and in Bungoma county and identifies factors facilitating and inhibiting progress in Bungoma county for wider learning in Kenya and other similar countries.

## Methods

A mixed method approach using quantitative and qualitative methodologies was used to collect and analyse data. The quantitative analysis was conducted first and Bungoma was identified as a high performing county within Kenya on perinatal death reporting and review. Based on this, qualitative methods helped examine factors in Bungoma influencing the implementation of perinatal death reviews, with a focus on actions taken.

The quantitative findings are based on perinatal data downloaded from the Kenya DHIS2 in August 2018 to analyse trends in perinatal death reporting and reviews between 2014 and 2017. Data on the number of deaths reported was obtained from the Ministry of Health’s integrated summary report for reproductive and child health form. Data on perinatal death reviews was obtained from the perinatal death review form. We first analysed national level data from Kenya, and then stratified by county to review findings in Bungoma county. All analysis was conducted based on the year in which the death occurred. Deaths were excluded from the analysis if there was missing data such as date of death.

For the qualitative analysis, focus group discussions were conducted with MPDSR committee members (approximately 12–15 participants per MPDSR committee) in three purposefully selected hospitals in Bungoma county where the majority of perinatal deaths occur. After the initial analysis of transcripts, further information was required to dig deeper into the perinatal aspects of the MPDSR cycle. Additional interviews were conducted with the Health Records Information Officer (HRIOs) in one of these hospitals, while the maternity in-charge together with the HRIO were interviewed in another of the hospitals initially selected. Two additional hospitals were identified to gather more insights where the MPDSR committee was interviewed at one hospital and the HRIO at another hospital. Finally, a facility nursing officer in-charge was interviewed at a health centre. In total, 5 hospitals and 1 health centre were part of the sample. In the second round of interviews, either the MPDSR committee or individuals were purposively selected based on the type of information required and to target different types of facilities. A thematic analysis of transcripts was conducted to draw out codes on enabling and inhibiting factors to reviewing and acting on perinatal deaths, as well as the types of actions taken and at what level to affect quality of care improvements. These codes were analysed using Dedoose software where codes were re-grouped back into themes. All discussions were recorded and transcribed with consent provided from all participants. Focus group discussions and interviews have been anonymised to protect the identify of individual health facilities and potential cases.

## Results

### Quantitative analysis

A total of 88 facilities in Bungoma implement MPDSR, of which 42 facilities have been supported by the MANI project. The number of perinatal deaths that occur in each facility are reported in the DHIS2. It is important to note that the DHIS2 only captures those deaths that occur in a facility, and it is likely that not all facility-based deaths are reported to the platform or reviewed. Table 1 shows the numbers of perinatal deaths reported in Kenya and Bungoma county between 2014 and 2017, as per the DHIS2. The data shows the number of perinatal deaths occurring each year have decreased between 2014 and 2017, in Kenya as well as in Bungoma county. In 2017, a total of 28,614 perinatal deaths were reported nationally, and of these, 891 were reported from Bungoma County. Over the years, the number of perinatal deaths in Bungoma as a proportion of total perinatal deaths in Kenya has increased slightly; although the number itself has decreased.

**Table 1:** Number of perinatal deaths reported in Kenya and Bungoma between 2014 - 2017

Number of perinatal deaths	2014	2015	2016	2017
Kenya	36,598	32,511	32,743	28,614
Bungoma County	1042	949	984	891
Number of perinatal deaths reviewed in Bungoma county	2	131	600	524
Number of Perinatal deaths reviewed in Bungoma as a proportion of total perinatal deaths reported in Bungoma	0%	14%	61%	58.8%
Perinatal deaths reported in Bungoma as a proportion of total perinatal deaths reported in Kenya	2.85%	2.92%	3.01%	3.11%

Table 2 presents the number of perinatal deaths reported and reviewed in Kenya in 2017 and shows perinatal death reviews were conducted in just 13 of the 47 counties. The table also demonstrates that of the 28,614 perinatal deaths that occurred in the country in 2017 (i.e. a year after the perinatal death review system was introduced), only 3.6% (1027) of deaths were reviewed.

**Table 2:** Perinatal deaths reported and reviewed across counties in Kenya (2017)

County	Number of deaths reported	Number of deaths reviewed	Proportion of deaths reviewed	Proportion of the total deaths in Kenya	Proportion of all perinatal death reviews in Kenya
Bungoma	**891**	**524**	**58.8%**	**3.1%**	**51.0%**
Kakamega	1012	234	23.1%	3.5%	22.8%
Nakuru	1411	195	13.8%	4.9%	19.0%
Kisumu	841	19	2.3%	2.9%	1.9%
Migori	556	17	3.1%	1.9%	1.7%
Kisii	675	10	1.5%	2.4%	1.0%
Lamu	142	9	6.3%	0.5%	0.9%
Wajir	320	5	1.6%	1.1%	0.5%
Homabay	543	5	0.9%	1.9%	0.5%
Nyandarua	461	3	0.7%	1.6%	0.3%
Baringo	328	3	0.9%	1.1%	0.3%
Kajiado	440	2	0.5%	1.5%	0.2%
Nairobi	4498	1	0.0%	15.7%	0.1%
Kenya	**28,614**	**1027**	**3.6%**		

**Fig. 1:**
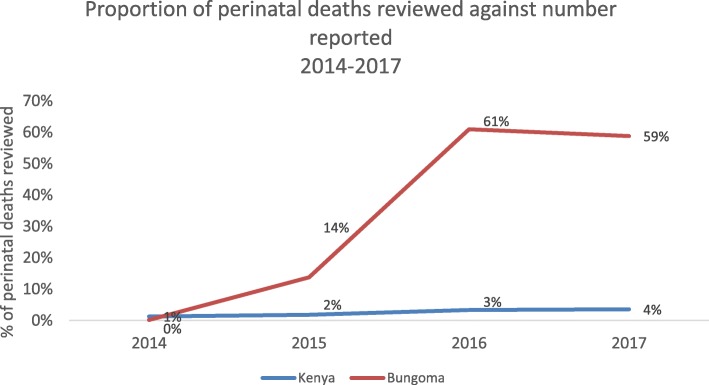
Proportion of perinatal deaths reviewed against the number reported in Kenya and Bungoma (2013–2017)

A similar analysis of the proportion of perinatal deaths reviewed from 2014 to 2017 shows an approximate one percentage point increase each year at the national level (Fig. 1). In Bungoma county, however, the proportion of perinatal deaths reviewed has been consistently higher than the national level, with 14% of deaths having been reviewed in 2015 even prior to the formal introduction of ‘P’ in the MDSR system. In 2017, 59% of the perinatal deaths reported in Bungoma county were reviewed, outperforming all other counties in Kenya. This amounts to 51% of all the perinatal deaths reviewed in Kenya in 2017 (see Table 2).

**Fig. 2:**
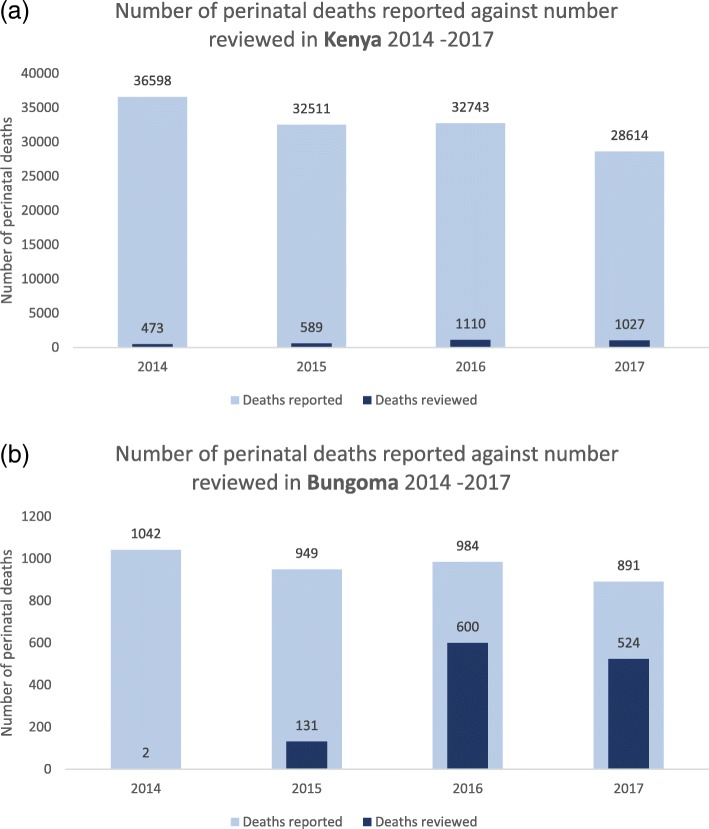
Number of perinatal deaths reported and reviewed in (**a**) Kenya and (**b**) Bungoma (2014–2017)

Figure 2a and b confirm that both Kenya and Bungoma have increased the proportion of perinatal deaths that are reviewed, however Bungoma has done so at a much higher rate. In Bungoma, the proportion of perinatal deaths reviewed in 2017 decreased by two percentage points from the previous year, as a consequence of the widespread health provider strikes in 2017. A number of secondary and tertiary facilities where comprehensive emergency obstetric and newborn care are provided were shut down for some months and all services and record-keeping were affected as a result.

The findings from the DHIS2 analysis highlight that the MDPSR system was quickly established and the practice of undertaking reviews is being embedded across facilities in Bungoma County in a stronger manner, as compared to other counties across Kenya. With over 50% of perinatal deaths currently being reviewed, Bungoma is already leading the way in Kenya, but as with other sections of the health management information system, this too presents challenges with data quality. Incomplete, inaccurate or inconsistent records on identification of type of death, underlying causes of death and details on case notes still need to be addressed as the system gets institutionalised [13].

### Qualitative analysis

As shown above, Bungoma county is out-performing the rest of Kenya in reporting and reviewing perinatal deaths. The qualitative analysis examines factors facilitating an enabling environment for emphasising the ‘P’ in MPDSR.

#### Improvements to perinatal death reporting and review

All facilities have been reviewing maternal deaths, but very few were conducting perinatal death reviews. Awareness and training was a critical first step in mobilising health staff to attach prominence to and conduct perinatal death reviews:

*Mostly we were reviewing maternal deaths not perinatal deaths. When we were trained that is the time we started perinatal death reviews, the training was helpful … It was not easy to mobilise staff to participate in this activity. We were able to hold facility meetings, we educated them on how important it was and so they were able to understand … We were given knowledge and we were able to identify avoidable causes, service delivery and quality gaps and through the review we have improved in service delivery, quality of work and team work - Health Centre, Individual Interview*

Having the appropriate perinatal death review reporting templates in place at the facility level which align with national online forms (i.e. DHIS2) was a key challenge facing all health facilities. The paper forms used by the MPDSR committees in facilities was different from the online national DHIS2 form. The harmonisation of the facility level hardcopy tool with the national online form paved the way in improving efficiencies in reporting, which was otherwise burdensome. In addition, including the HRIOs as members of the review committees helped ensure information is uploaded in a timely and accurate manner.

*The reporting tools are now available. The initial forms were not comprehensive enough, I remember [an external partner - MANI} helping us to align DHIS reporting with the review form, without this I’m not sure the review would be happening now. It is good that the HRIOs were involved in the training. It is easier for the facilities to know how many deaths to be reviewed in advance. The presence of the HRIO in the review meetings ensures uploading is done immediately after the review because they know what was discussed - Hospital A, Individual Interview*

The volume of perinatal deaths compared to maternal deaths has made it a challenge for MPDSR committees to review all perinatal deaths. When perinatal death reviews began, initially only a few were reviewed, however facilities have made progress in increasing the proportion reviewed, based on changes to how the reviews occur and the frequency of meetings:

*For perinatal deaths, it was low, below 50% but now we are above 95% for review of perinatal and 100% for maternal deaths. 100% review for perinatal deaths is unrealistic, we cannot manage to review it here, otherwise it will mean having permanent staff whose work is to just review - Hospital A, Individual Interview*

*There were many perinatal deaths at one time and there was a challenge reviewing them. Monthly meetings helped to clear the backlog and through the MPDSR interventions, the mortalities have equally reduced … Sometimes we had to share in groups when the perinatal deaths were many, each group would then present to the whole committee and together we discussed emerging issues - Hospital E, Individual Interview*

A key factor influencing participation in and continuity of perinatal reviews is ensuring the focus remains on learning and improving quality of care rather than on placing blame or engaging in punitive measures. Staff attitudes have therefore shifted from apprehension to positivity in using perinatal reviews to learn, share experiences and take action:

*We actually embraced teamwork and empowered everybody involved in maternal and newborn health services, not blaming others but improving quality. Whenever one had a mortality, we told them to freely share information for us to review - Health Centre, Individual Interview*

#### Implementation of actions to improve perinatal outcomes

The presence of a strong clinical team that was goal oriented and focused on implementing findings from perinatal death review meetings has facilitated implementation of perinatal actions. The sharing of responsibility for implementing actions, not only among those within the committee but also to those in a position to influence implementation are important ways to foster teamwork, close the loop on the review cycle and strengthen accountability:

*Before we found that clinical service providers were not on the same page, but with MPDSR we are able to work as a team, we have collective team work. When people team up to do things with one goal we have attained our goals. – Hospital A, MPDSR Committee*

*Initially when MPDSR started it took time to sensitize people and for people to understand why it is important that we review perinatal deaths. It worked for us when we brought the administrator because when we come up with action points, we give them the minutes and they help us implement – Hospital B, MPDSR Committee*

Perinatal death review actions are regularly tracked by the MPDSR committee with feedback provided on the status of each recommendation. This process facilitates a shared sense of accountability, with those individuals assigned actions reporting back to the committee on progress:

*After making the recommendation we usually have the timeframe that we are going to follow and ensure that the recommendations have been done, during the subsequent MPDSR meeting we usually give feedback from the previous meeting. - Hospital C, MPDSR Committee*

The inherent value of perinatal death reviews is the observable changes to service delivery and quality of care that resulted from the implementation of recommendations from the reviews. The actions implemented vary from improved knowledge through continual medical education, better prevention and case management practices, strengthened referral systems due to better communication, and influencing key decision-makers to obtain needed equipment and supplies. Importantly, these actions are within the control of and implemented within the facility setting, reinforcing confidence among committee members that some, if not most of the recommendations emerging from perinatal death reviews can be implemented directly by facility staff.

*Since we have identified that our biggest cause of neonatal deaths is pre-maturity and its complications, neonatal sepsis and birth asphyxia, we have tried to put interventions in place to tackle each one of them. Implementation of kangaroo mother care is now universal, everybody working here knows that preterm and low birth weight babies require kangaroo mother care. In preventing birth asphyxia at the maternity, they are now more focused, not just on delivering a baby, but the quality of the baby that they deliver. For sepsis, last month we were able to get a blood culture machine– Hospital B, MPDSR Committee*

One of the key perinatal death review actions that also cuts across maternal survival is improved case history and patient tracking through better documentation. Accurate and detailed documentation is an important means through which the continuum of care and case management of both mother and the status of her baby from one facility to another has greatly improved.

*We had so many problems with documentation, it has improved because of MPDSR. When you look at the case notes if they are not documented well, then the case will not get anywhere and you cannot get any concrete way forward. When we have good documentation, you realize that there is that continuity of care so it has reduced perinatal deaths - Hospital A, MPDSR Committee*

*The first thing we noted with stillbirths is that mothers were not monitored using the partograph. It was agreed in one of the meetings for nurses to use the partograph in monitoring labour. After that, nurses started using it and cases that were as a result of ignorance or poor management of mothers in maternity started reducing - Hospital E, Individual Interview*

Supportive supervision from sub-county and facility management levels have helped establish successful perinatal death reviews, which have also been used to advocate for the implementation of actions at the county level:

*Periodic support supervision has been very helpful and it reminds us to do what is needed. The sub-county has been keen in petitioning our claims to the county especially with improving staffing. Distribution of essential items like pharmaceuticals … All this I would say have contributed to the success of perinatal death reviews - Health Centre, Individual Interview*

Communication and feedback of learning between referral and referring facilities has also led to improvements in the management of preterm babies. A factor contributing to better communication is that most referring facilities have representatives that are part of the MPDSR committee at sub-country or county level which enables a shared understanding of causes and solutions to prevent further perinatal deaths:

*This had a great and positive influence in terms of referral, at least now the facilities are calling beforehand, they are accompanying the clients with a partograph for further management. Babies that maybe are preterm, they are referred and implementing kangaroo mother care - Hospital B, MPDSR Committee*

*During the sub-county MPDSR meeting, the other facilities in the sub-county are normally in that meeting so if your facility referred a patient without a partograph they are all told from that meeting and they are able to improve from that – Hospital C, MPDSR Committee*

Actions from perinatal death reviews have recommended stronger linkages with the community to help identify high risk mothers and tackle the first delay in a woman’s decision to seek antenatal care to improve outcomes for both mother and baby. Encouraging facilities and communities to make timely decisions and respond appropriately with minimal delay has helped strengthen the referral system.

*When we do these reviews, and we find that first delay is a problem that is increasing maternal and neonatal death, then we empower the community with knowledge on how to educate the mothers to attend antenatal care early and at least attend all the clinics for management of dangers signs. That’s how we are improving so that there are no maternal or perinatal deaths in the community – Hospital C, MPDSR Committee*

#### Feedback of perinatal death review findings at various levels

The extent to which feedback from perinatal death reviews is communicated to other health staff and management within the facility, to the community and to higher officials within the health system influences the extent to which recommendations are implemented. Feedback of perinatal death review recommendations is provided in some instances to communities, particularly in areas in which the public can co-develop solutions or address the issue:

*We normally give feedback to the community, through our monthly meeting. If we had a problem or something that I am supposed to communicate to them, we share together and get a way forward. And that’s how we don’t have prenatal or maternal deaths in the community – Hospital A, MPDSR Committee*

Efforts are made across facilities to share perinatal death review recommendations with other health staff and departments. However, due to time pressures, competing tasks and staff availability or turnover, feedback is not always provided to all who need it. There are cases where some in the department are unaware of what transpired during the perinatal death review or what needs to be changed:

*We try our best to give feedback to all the service providers. Not all staff are on duty, so when you give feedback, you would expect the other person also to pass it on, but you will have a few people in the department who really don’t know what is happening – Hospital B, MPDSR Committee*

Feedback of findings from the perinatal death reviews to referring facilities is provided when a gap in care requires their attention. In some cases, if sub-optimal care has been provided, there is immediate communication with the referring facilities even before the perinatal death review has taken place:

*We do it immediately, we don’t even wait for a review meeting, like over the last two weeks we were getting babies in bad condition from a referring facility, we just tell them immediately because they get a sense of that case if you give feedback immediately rather than waiting - Hospital B, MPDSR Committee*

Sharing recommendations from perinatal death reviews with management within the facility as well as at sub-county and county levels is important to support MPDSR committees in implementing actions beyond their control and to help unblock systemic bottlenecks that can help improve perinatal outcomes across the health system.

*We give feedback to the medical superintendent because most of the action points require the input of the administration for implementation. We also give feedback to the sub counties, especially if you have specific cases where there was an omission, a delay in referral or mismanagement before referring the patient - Hospital B, MPDSR Committee*

A key challenge faced is the inability to act on all perinatal death review recommendations either due to a lack of financial resources or the inability to implement actions outside the sphere of control of the facility setting.

*For things within reach of hospital capacity, 90% is done, but anything that needed sub-county and county effort, not much has been achieved … We used to like to go through the previous action points one by one and whoever was responsible would give feedback, but again anything beyond the hospital would not be done. Over time, this can be discouraging - Hospital E, Individual Interview*

Some facilities however are concentrating on the implementation of actions which are within their control. This has proved motivating with observed improvements in perinatal survival:

*We know that deaths are notifiable and there is a need to review. With the reduction in perinatal deaths over time, this has encouraged us as we presumed care has improved. We are able to come up with simple doable actions that we could just do ourselves first rather than wait for external support - Hospital D, MPDSR Committee*

## Discussion

Bungoma county has outperformed the rest of Kenya in reporting perinatal deaths into the national system and conducting reviews. Factors contributing to the success of perinatal death reporting and review in Bungoma include: harmonisation of the facility based perinatal reporting tools with the national level; prioritising the need to document and report mortalities; tailoring continual medical education and supportive supervision visits to the needs identified from the reviews; and better documentation and referral processes. Supportive management and administrative staff have also helped drive forward implementation of actions and increased health staff motivation to reduce perinatal deaths and improve quality of care.

As in other low and middle income settings, perinatal death reviews are more effective when used to inform quality of care improvements [11], which has been a clear focus of MPDSR committees in Bungoma county. Importantly, the perinatal death review process in Bungoma has ensured, where possible, that recommended actions are implemented, thus working to complete the full MPDSR cycle from reporting, review, monitoring and action to affect change [6, 10]. In cases where the implementation of perinatal death review recommendations requires management input, MPDSR committees have used different strategies to advocate by encouraging management to participate in reviews or compelling decision makers to prioritise and allocate required resources [6]. The implementation of perinatal death review recommendations helped change service delivery practices in Bungoma such as improving skill gaps, communicating preventative practices to referring facilities and improving documentation and referral processes. Identifying preventable factors leading to perinatal deaths can have positive outcomes in reducing such deaths if solutions are implemented locally, as is the case in Bungoma [11].

Effective, successful and sustainable implementation of perinatal death reviews is founded on phases of the change cycle [15], which has been followed in Bungoma: 1) The *drivers of change:* MPDSR committees have demonstrated leadership in ensuring perinatal death review meetings are learning exercises. This along with collective team work and assigned responsibility and accountability for actions have motivated staff to focus on implementing perinatal death reviews [16–18]. Having perinatal death review meetings on their own does not result in quality of care improvements, however the assignment of responsibilities for implementing and monitoring actions has facilitated the change process [15, 16]. 2) *Clinical outreach visits and supervisory activities:* MPDSR committees provide immediate feedback to referring facilities either before the perinatal death review has taken place when there is an urgent quality of care gap that requires attention or after the perinatal death review when improvements to quality could be made in the future. 3) *Perinatal death reviews and feedback meetings are institutionalised* within comprehensive MPDSR meetings in Bungoma. The quality of data feeding into reviews has helped ensure appropriate and localised solutions are developed and communicated to the right level [11]. 4) *Communication and networking at different levels of the health system:* Perinatal death review findings are used to advocate to facility management and those at sub-county and county level to implement actions that require resources to be mobilised. Unlike other studies in Senegal and Uganda [17, 19], findings from perinatal death reviews in Bungoma are shared with communities so that communities have greater awareness of and can act on recommendations within their control [11, 13, 20].

The implementation of perinatal death review actions should be done across different areas of the health system to affect wider change [15]. The *policy* area is where the implementation of actions from perinatal death reviews in Bungoma has faced the most challenge, as political and funding decisions are required that fall beyond the sphere of control of health facilities. More success has been made in the *administration* area, where specific recommendations from perinatal death reviews requiring health facility management decision-making have been communicated and acted upon. In these cases, some heath facility managers in Bungoma have been able to prioritise or shift resources for the implementation of actions. The *clinical practice and training* area is where most strides have been made, as these actions fall within the control of facilities in Bungoma, such as ensuring health staff training and supportive supervision on topics such as Kangaroo care are based on skill gaps identified by the perinatal death review. As in Malawi [21], health staff in Bungoma felt motivated by reviews when observable declines were noted in perinatal deaths. Our study confirms other findings emphasising the importance of actions implemented in influencing the conduct and value of perinatal death reviews [17, 22].

To address the challenge of implementing perinatal death review actions beyond the control of health facilities in Bungoma, holding health mangers, administrators and others at different levels of the health system accountable for implementation can be encouraged by setting key performance targets [16]. Defining targets to reach impact indicators and outcomes on perinatal survival at sub-national and facility level can contribute to more sustainable perinatal death review and response systems [15].

## Conclusion

Factors influencing or hindering perinatal death reviews and response in Bungoma can strengthen wider scale-up across Kenya and beyond. Our study responds to the sparse literature by demonstrating how perinatal reviews, linked to the wider MPDSR system, are a tool to optimise quality of care. It confirms that successful implementation of perinatal death reviews requires clear delineation of roles and responsibilities for action and that the implementation of those actions are routinely monitored to track implementation progress. As in other low-income settings, Bungoma county has demonstrated that in Kenya, perinatal death reviews can be effectively implemented and sustained, through a focus on learning, solution-oriented responses, influencing those in a power to act, accountability for results, and observable quality of care improvements.

## Data Availability

The datasets generated and/or analysed during the current study are not publicly available to maintain confidentiality of individuals interviewed at the hospital and health centres but are available from the corresponding author on reasonable request.

## Abbreviations

DHIS: District Health Information System
MANI: Maternal and Newborn Health Initiative
MPDSR: Maternal and Perinatal Death Surveillance and Response
SDG: Sustainable Development Goal
